# A comparative study of two liquid-based preparation methods: membrane-based and sedimentation in fine needle aspiration cytology diagnosis in thyroid nodules

**DOI:** 10.1186/s12957-020-1787-1

**Published:** 2020-01-16

**Authors:** Juan Zhao, Xiaofei Yao, Chunjiao Song, Cheng Wang

**Affiliations:** 1 Department of Pathology, Shaoxing People’s Hospital (Shaoxing Hospital, Zhejiang University School of Medicine), No. 568 Zhongxing North Road, Shaoxing, 312000 China; 2 Medical Research Centre, Shaoxing People’s Hospital (Shaoxing Hospital, Zhejiang University School of Medicine), Shaoxing, 312000 China

**Keywords:** Membrane-based liquid-based preparation technique, Sedimentation liquid-based preparation technique, Thyroid fine needle aspiration cytology (FNAC), Papillary thyroid carcinoma (PTC)

## Abstract

**Background:**

As thyroid fine needle aspiration (FNA) shows a certain limitation in the diagnosis of conventional smears, novel approaches like liquid-based cytology (LBC) have been gradually applied recently. Studies have shown the difference between the conventional smears (CSs) and liquid-based smears on fine needle aspiration cytology (FNAC) diagnosis, but the impacts of different liquid-based preparation (LBP) methods, including membrane-based and sedimentation, on diagnosis are still not clear. In this study, the effects of liquid-based smears prepared by different methods on the cytological interpretation were studied.

**Methods:**

A total of 221 thyroid liquid-based FNAC cases from January 2017 to October 2018 were collected. We retrospectively studied and compared the effects of the membrane-based and sedimentation LBP methods through The Bethesda System for Reporting Thyroid Cytopathology (TBS) diagnosis and risk of malignancy assessment. Besides, we made an evaluation on the diagnostic differences in the effects of different preparation methods on the cell morphology and tissue structure of papillary thyroid carcinoma (PTC) for more accurate FNAC diagnosis.

**Results:**

Among the 221 cases reviewed, membrane-based method was applied in 153 cases and sedimentation in 68 cases. According to the diagnostic criteria of 2017 TBS, TBSVI and TBSV thyroid could be cytologically diagnosed by membrane-based (49.0% (75/153) and 25.5% (39/153)) and sedimentation (52.9(36/68) and 25(17/68)) methods, and both were confirmed as PTC through histopathological diagnosis after operation, with the malignancy degree as high as 100%. In addition, of the 30 cases that were diagnosed as TBSIII thyroid nodules with the membrane-based method, 15 cases were pathologically malignant after an operation, with the malignancy degree of 50% (15/30), while that in 11 cases using the sedimentation method was 45.4% (5/11). PTC could be detected in both the TBSIV and TBSII thyroid nodules diagnosed by membrane-based method, with the sensitivity of 87.0% (114/131) lower than that by sedimentation method (91.4% (53/58)), showing the lower consistency with the histopathological result (*K* = 0.635 vs *K* = 0.757). Among the membrane-based smears, 23.5% (36/153) had fewer follicular epithelial cells, 55.6% (20/36) of which were considered to be suspicious for PTC from cell karyotype and tissue arrangement. While among the sedimentation smears, 16.2% (11/68) had fewer follicular epithelial cells, and 63.6% (7/11) was suspicious for PTC. In 72.5% (95/131) membrane-based smears of PTC, the papillary and swirling structures were not obvious, showing as crowded syncytial cell masses, while in 55.2% (32/58) sedimentation smears, both structures were visible with obvious three-dimensional papillary structure, and the fibrovascular axis still remained.

**Conclusion:**

LBP technique is feasible for FNAC diagnosis, and the sedimentation shows more advantages, like higher PTC detection rate and good consistency with postoperative histopathological diagnosis. A clear understanding of the subtle differences in the effects of membrane-based and sedimentation methods on the cell morphology and tissue structure could be conducive to the definitive diagnosis of PTC before operation.

## Introduction

With the application of ultrasound-guided diagnosis for thyroid nodule screening becoming much more popular, ultrasound-guided fine needle aspiration (FNA) technique emerged. Cytological diagnosis is particularly important for the preoperative evaluation on thyroid nodules. Therefore, the way of obtaining more specimens and preserving cell morphology well to make an accurate cytological interpretation or further molecular detection has become the present focus. For operators, conventional smear technique is much more difficult to master. Not only the rapid movement is required, but also the smear thickness should be as uniform as possible. Especially for the specimens with massive amounts of blood, even experienced operators may also cause cell loss and degeneration when smearing. In the domain of non-gynecology, liquid-based preparation (LBP) technique can overcome the above difficulties. Based on this technique, all the cells obtained by aspiration could be directly transferred into a bottle containing preservation solution and then well distributed on a certain range of a glass slide through membrane-based or sedimentation LBP technique.

The differences between the conventional smears and liquid-based smears in the evaluation of FNAC diagnosis in thyroid nodules have been compared in many studies [1–3], showing the superiority and reliability of the LBP technique. However, the effects of different LBP methods, including membrane-based and sedimentation, on diagnosis are still not clear. Moreover, the differences in preparation principles, the composition of the preservation solution, and the scopes of microscopies all can affect the cell interpretation. In the present study, the effects of different LBP methods on the cytological diagnosis were studied. Meanwhile, we analyzed the results of FNAC diagnosis and postoperative histopathology diagnosis of 221 cases from January 2017 to October 2018 and compared the effects of the membrane-based and sedimentation LBP methods through The Bethesda System for Reporting Thyroid Cytopathology (TBS) diagnosis and risk of malignancy assessment. Moreover, in order to make the liquid-based FNAC diagnosis accurate, the diagnostic differences in the way of different preparation methods on the cell morphology and tissue structure of papillary thyroid carcinoma (PTC) were also evaluated.

## Materials and methods

### Clinical data

Pathological files of 221 cases diagnosed by liquid-based FNAC in thyroid nodules from January 2017 to October 2018 in our hospital were collected. All of them were histopathologically diagnosed after operation.

### Methods

Thyroid nodules with indications to puncture were processed for ultrasound-guided FNA using a 21G puncture needle. Cells obtained were transferred into preservation bottles and then prepared to liquid-based smears for follow-up TBS diagnosis.

The mechanism of the membrane-based method refers to that cells are transferred onto a glass slide forming a diagnostic area of 20 mm in diameter using a membrane-based Cell Prep Plus LBC Processor (TinPrep2000TM, Hologic Co., USA). While the sedimentation method is based on a Sedimentation Cell Prep Plus LBC Processor under the LBP system (LBP-2601, Anbiping, Guangzhou), with cells automatically sedimented onto a glass slide forming a diagnostic area of 13 mm in diameter. Both two techniques end with HE staining.

In this study, we firstly retrospectively studied the consistency between the results of FNAC diagnosis based on different preparation methods and histopathological diagnosis. Next, the effects of different preparation methods on TBS diagnosis and malignancy assessment were compared and analyzed. Finally, the difference in these two preparation methods on the morphology and tissue structure of thyroid follicular epithelial cells was evaluated.

### Diagnostic criteria

According to the diagnostic criteria of 2017 TBS [4], thyroid nodules classified as TBSVI or TBSV were defined as positive, while those classified as other TBS classifications were negative. Besides, nodules histopathologically diagnosed to be malignant were positive, whereas benign were negative.

### Statistical analysis

SPSS 19.0 software was used for statistical analysis. The relationship between cell count obtained by ultrasound-guided FNA and clinical characteristics was analyzed in chi-squared test. Paired *χ*^2^ test was conducted to validate whether liquid-based FNAC diagnosis based on different preparation methods and histopathological diagnosis could obtain consistent results, and the kappa value was calculated. Kappa ≥ 0.75 showed good consistency; 0.75 > kappa ≥ 0.4 showed the general consistency; and kappa < 0.4 showed poor consistency. *P* < 0.05 was considered statistically significant.

## Results

### Clinical data

A total of 221 cases from January 2017 to October 2018 were collected, including 43 males and 178 females, aged 20–76 years. Clinical characteristics included the nodule location, nodule size, and ultrasound blood flow signal (yes/no) as listed in Table 1. Among the 153 cases with membrane-based method, 74.5% (114/153) cases were positive and 131 cases were histopathologically diagnosed as PTC after operation. While of the 68 cases with sedimentation method, 77.9% (53/68) cases were positive and 58 cases were confirmed as PTC (Tables 2 and 3).

**Table 1:** Clinical characteristics of the 221 cases made by different preparation methods

Preparation methods/clinical characteristics	Nodule location			Maximum diameter		Ultrasound blood flow signal	
	Left	Right	Isthmus	≥ 1 cm	< 1 cm		
						Yes	No
Membrane-based	77	69	7	39	114	70	83
Sedimentation	29	38	1	29	39	25	43

**Table 2:** TBS diagnosis results of the 221 cases made by different preparation methods

TBS diagnosis	Membrane-based (%)	Sedimentation (%)
TBSII	6 (3.9%)	4 (5.9%)
TBSIII	30 (19.6%)	11 (16.2%)
TBSVI	3 (2.0%)	0 (0%)
TBSV	39 (25.5%)	17 (25%)
TBSVI	75 (49.0%)	36 (52.9%)
Total	153	68

**Table 3:** PTC diagnosis results of the TBS diagnosed cases made by different preparation methods

	TBSII	TBSIII	TBSVI	TBSV	TBSVI	Total
Membrane-based	1	14	2	39	75	131
Sedimentation	0	5	0	17	36	58
Total	1	19	2	56	111	189

### FNAC diagnosis based on membrane-based LBP method

In all, 153 samples prepared by membrane-based LBP technique were collected (Table 2). According to the TBS diagnosis, TBSVI malignant papillary carcinoma was found in 75 cases, TBSV suspicious for malignancy in 39 cases, TBSIV suspicious for a follicular neoplasm in 3 cases, TBSIII atypical follicular epithelial cells of undetermined significance in 30 cases, and TBSII benign lesions in 6 cases. Thereinto, cases that classified as TBSVI and TBSV were all confirmed to be PTC through histopathological diagnosis, with 100% in consistency; 66.7% (2/3) TBSIV cases were confirmed to be PTC; 50% (15/30) TBSIII cases were histologically confirmed to be malignant, including PTC diagnosed in 14 cases and medullary carcinoma in 1 case; and 1 TBSII case was confirmed to be PTC. On the whole, the sensitivity of the membrane-based preparation technique for thyroid malignancy diagnosis was 86.4% (114/132), while that for PTC diagnosis was 87.0% (114/131) (Table 3). Through analysis, we found that the malignancy degree of TBSIV thyroid nodules was 66.7% (2/3), while that of TBSIII and TBSII was 50% (15/30) and 16.7% (1/6), respectively. Statistical analysis showed that the kappa value was 0.635, and there had a difference between the cytological interpretation based on the membrane-based LBP technique and the postoperative histopathological diagnosis (*P* = 0.000) (Table 4)

**Table 4:** Consistency analysis in FNAC and corresponding histopathological diagnosis of 153 membrane-based cases

Membrane-based	Histopathology		*χ*^2^ value	*K* value	*P* value
	Negative	Positive			
Negative	21	18	71.150	0.635	0.000
Positive	0	114			

### FNAC diagnosis based on sedimentation LBP method

Totally, 68 samples prepared by sedimentation LBP technique were collected (Table 2). Based on the TBS diagnosis, TBSVI was found in 36 cases, TBSV in 17 cases, TBSIII in 11 cases, and TBSII in 4 cases. Thereinto, in agreement with the membrane-based cases, TBSVI and TBSV cases were all confirmed to be PTC through histopathological diagnosis, with 100% in consistency. In addition, 5 TBSII cases were histologically confirmed to be PTC. On the whole, the sensitivity of the sedimentation method for PTC diagnosis was 91.4% (53/58) (Table 3). Besides, we could observe that the malignancy degree of TBSIII thyroid nodules reached 45.4% (5/11). Statistical analysis suggested that there was no significant difference between the cytological interpretation based on sedimentation LBP technique and the postoperative histopathological diagnosis (*P* = 0.063), with a good consistency (*K* = 0.757) (Table 5).

**Table 5:** Consistency analysis in FNAC and corresponding histopathological diagnosis of 153 sedimentation cases

Sedimentation	Histopathology		*χ*^2^ value	*K* value	*P* value
	Negative	Positive			
Negative	10	5	41.625	0.757	0.063
Positive	0	53			

### Effects of membrane-based and sedimentation LBP methods on FNAC diagnosis

The effects of different preparation methods on the thyroid FNAC diagnosis were evaluated through many ways, including cell count, karyotype, and tissue arrangement, finding that both the membrane-based and sedimentation methods were feasible for FNAC diagnosis.

The cell count obtained through ultrasound-guided FNA is correlated with the experience and proficiency of operators, and also some clinical characteristics are involved in. In this study, the cell count was shown to be independent of thyroid nodule location and size, but associated with the ultrasound blood flow signals (*P* = 0.039) (Table 6).

**Table 6:** Relationship between the cell count and clinical characteristics

	Cell count		*P* value
	High	Low	
Nodule location			
Left	83	23	0.527
Right	86	21	
Isthmus	5	3	
Maximum diameter			
≥ 1 cm	118	35	0.318
< 1 cm	56	12	
Ultrasound blood flow signal			
Yes	81	14	0.039
No	93	33	

Conventional smears with visible follicular epithelial cell masses ≥ 6 are considered to be qualified; otherwise, they are considered to have less cell count. According to this standard, we found that in membrane-based smears, 36 cases had less cell count, among which 30 cases were confirmed as PTC after operation, including TBSIII thyroid nodules diagnosed in 10 cases (10/16) and TBSV in 20 cases (20/20). Despite the less cell count, PTC was highly suspected by cytological diagnosis in the way of karyotype and arrangement structure and confirmed by postoperative histopathological diagnosis, with 55.6% (20/36) in consistency. In sedimentation smears, 11 cases had less cell count, among which 9 cases were confirmed as PTC after an operation, including TBSIII diagnosed in 2 cases and TBSV in 7 cases, and the consistency reached 63.6% (7/11).

**Fig. 1:**
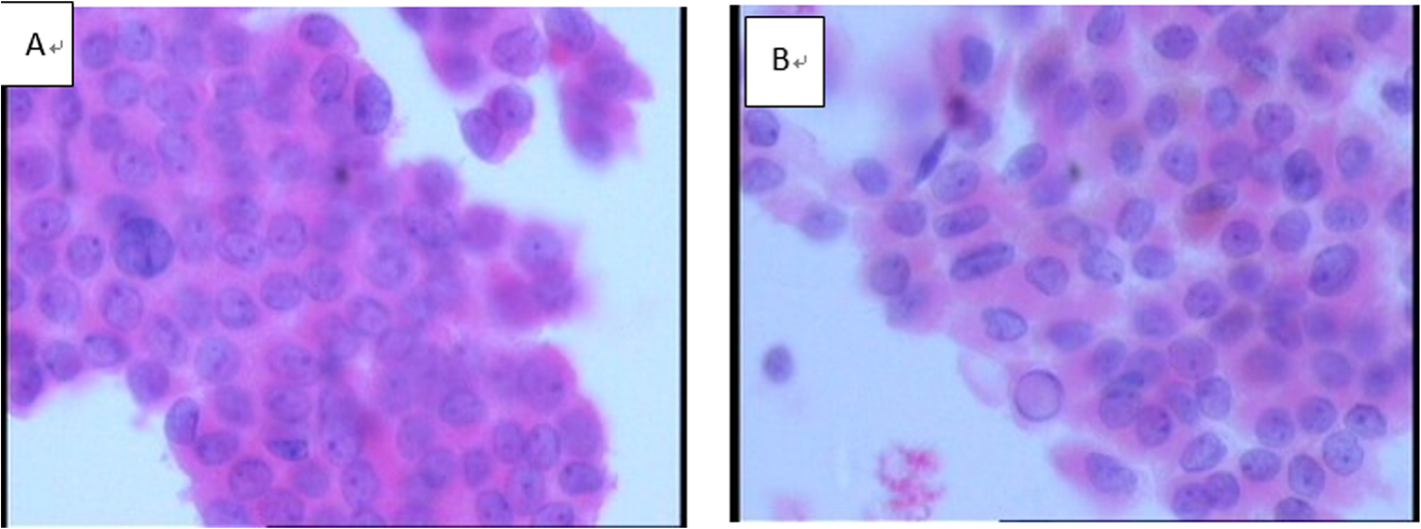
PTC nucleus characteristics like irregular karyotype, nuclei of glass appearance, nuclear grooves, inclusion bodies, and deviated small nucleoli all could be clearly seen. **a** Membrane-based smears. **b** Sedimentation smears(400*HE)

**Fig. 2:**
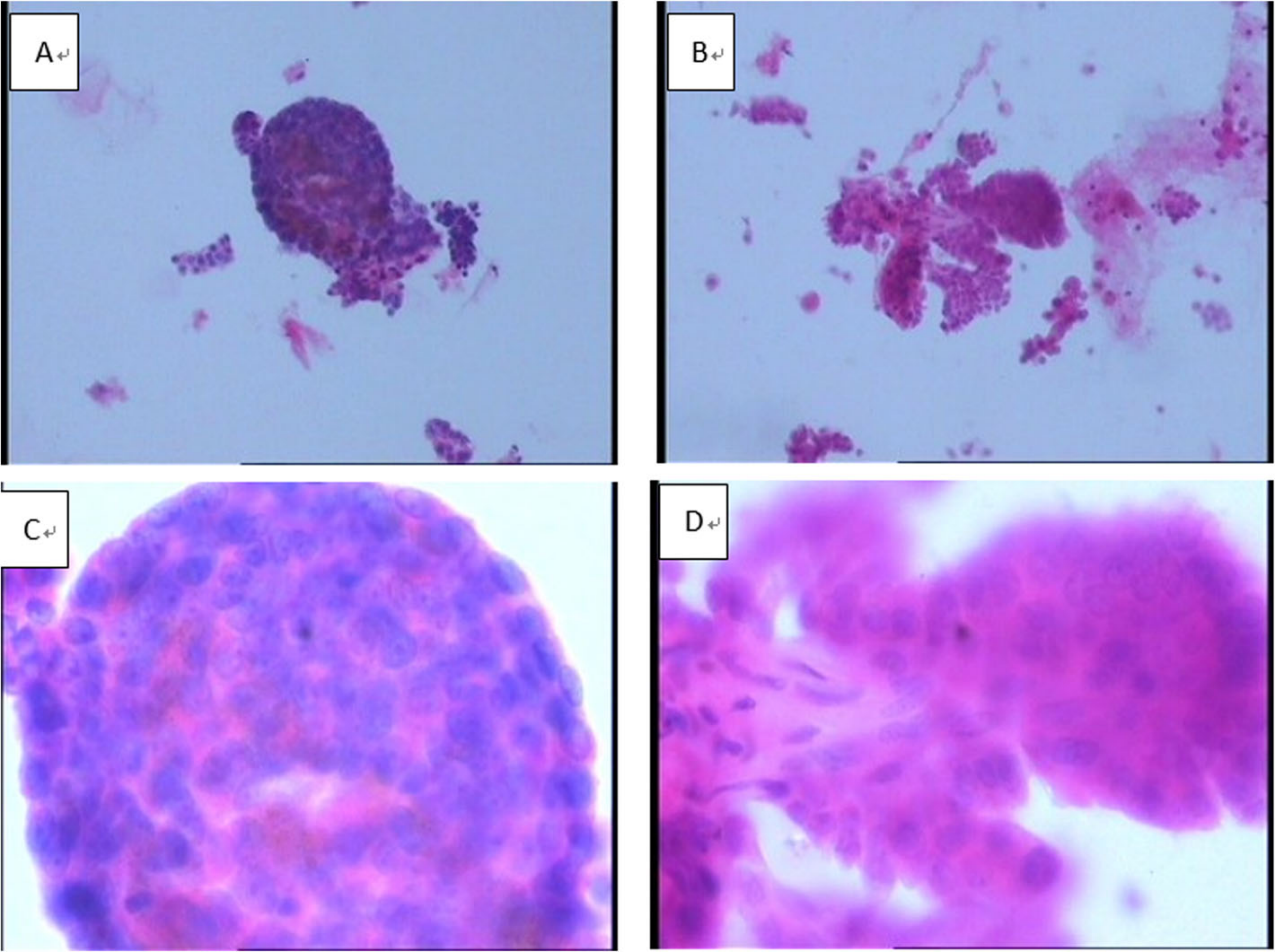
Papillary structure. **a** (× 100 HE) and **c** (× 400 HE), membrane-based smears with flat papillary tumor and crowded cell masses; **b** (× 100 HE) and **d** (× 400 HE), sedimentation smears with obvious three-dimensional papillary structure and fibrovascular axis

Cytological interpretation of PTC on conventional smears is based on nucleus characteristics (nuclei of glass appearance, nuclear groove, inclusion body, and deviated small nucleolus) and their arrangement modes (papillary, swirling, or crowded syncytial structures). In our study, both characteristics (Fig. 1) and arrangement modes of PTC nuclei could be clearly displayed by different LBP methods. Among the membrane-based smears, 72.5% (95/131) cases showed unobvious papillary or swirling structure but crowded syncytial cell masses, while among the sedimentation smears, both structures were visible with obvious three-dimensional papillary structure in 55.2% (32/58) cases, and the fibrovascular axis still remained (Fig. 2).

**Fig. 3:**
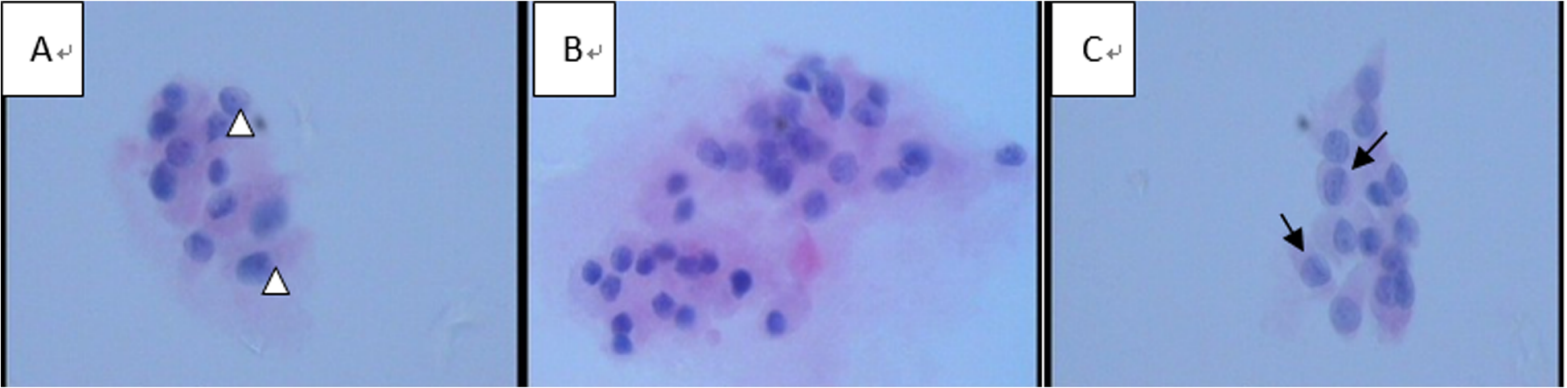
Tumor was cytologically interpreted as atypical follicular epithelial cells of undetermined significance and histologically confirmed as a benign lesion, with visible nucleus enlargement and round, fine staining, deviated small nucleoli (triangle), and nuclear grooves (black arrow) (× 400 HE)

Among the 14.4% (22/153) membrane-based smears that were histologically negative, 16 cases were cytologically diagnosed as TBSIII and above classes. Thereinto, 14 cases were diagnosed as TBSIII with visible nuclear enlargement and round, fine staining, deviated small nucleoli and nuclear grooves under an microscope (Fig. 3); 1 case was TBSV with irregular nuclear enlargement and crowded arrangement; and 1 case was TBSIV (suspicious for a follicular neoplasm) with slightly irregular nuclear enlargement and microfollicular structure. Among the 14.7% (10/68) sedimentation smears that were negative, 6 cases were diagnosed as TBSIII, including hyperchromatic nuclei, slightly irregular nuclear membranes, occasional inclusion bodies, and microfollicular epithelial structure showed in 50% (3/6) cases; 2 cases were diagnosed as nodular goiter with capsules wall cells characterized by rich cytoplasm and elongated nucleus; and the other 1 case was histologically diagnosed as chronic lymphocytic thyroiditis (LCT) accompanied by focal adenomatous hyperplasia, with irregular nuclear enlargement, nuclei of glass appearance, visible deviated small nucleoli, and microfollicular epithelial arrangement.

Much neutrophils and histocytes could be seen in the liquid-based smears of nodular goiter histologically and lymphocytes mixed with follicular epithelial cells appearing acidophilic degeneration in the smears of Hashimoto’s thyroiditis. However, these benign conditions could also be accompanied by malignant lesions, which would be mixed with peripheral normal follicular epithelial cells, thus bringing certain difficulties to cytological diagnosis.

## Discussion

LBP technique can be widely used in the preoperative thyroid FNAC diagnosis with many advantages, including well-preservation of cell morphology, less interference of blood cells, and cell aggregation for better observation, which can significantly improve the quality of the specimens made by ultrasound-guided FNA as well as the accuracy of cytological diagnosis. At present, the technique has been well applied in various hospitals, especially in primary hospitals. Although the accuracy of preoperative FNAC diagnosis on conventional smears can be improved aided with immunohistochemistry and gene detection [5], the combination of LBP technique and molecular pathology detection not only maximizes the use of puncture specimens, but also promotes a more accurate and comprehensive preoperative cytological evaluation of thyroid nodules.

Membrane-based and sedimentation methods are the major two ways of LBP technique in non-gynecological field. The mechanism of the former is that cells are evenly spread onto a glass slide forming a diagnostic area of 20 mm in diameter by air pressurization technique, while cells using the latter technique are uniformly sedimented onto a glass slide forming a diagnostic area of 13 mm in diameter through the method of density gradient centrifugation. There are several studies on the differences between the application of liquid-based smears and conventional smears in non-gynecological cytopathology [6–8], but few studies have shown the effects of these two LBP techniques on the preoperative thyroid FNAC evaluation. Elements like composition of the preservation solution, oscillation frequency, centrifugation speed, air pressurization technique, and density gradient sedimentation technique all can pose changes in cell morphology and tissue structure, directly affecting the cytological interpretation.

On the malignancy risk of TBSIII thyroid nodule, it is 10–30% indicated from the 2017 TBS, while in some studies, it is 31.2% [9] in the diagnosis of FNA conventional smears and up to 36.2% [10] in the diagnosis of sedimentation smears. In this study, a total of 221 cases were compared and analyzed from the results of the ultrasound-guided FNAC diagnosis under different LBP methods and corresponding histological diagnosis. We found that among the membrane-based and sedimentation smears, the malignancy risk of TBSIII thyroid nodules was as high as 50% (15/30) and 45.4% (5/11), respectively, and both were significantly higher than that occurred in conventional smears. Moreover, among the nodules that were cytologically interpreted as TBSIII with less cell count and confirmed as PTC after an operation, 62.5% (10/16) were microscopically with both atypical nuclei and structures of follicular epithelial cells in the membrane-based smears and 50% (2/4) in the sedimentation smears. Consistent with the study made by Gan et al. [11], despite the less cell count, the malignancy rate was increased in nodules with both atypical nuclei and structures of follicular epithelial cells. Moreover, membrane-based and sedimentation LBP techniques had a sensitivity of 87.0% (114/131) and 91.4% (53/58) for PTC diagnosis, respectively, and the preoperative cytological positive results based on these two techniques were completely consistent with the histopathological PTC diagnosis after operation. In addition, there was no significant difference between the sedimentation liquid-based cell interpretation and postoperative histopathological diagnosis, and the consistency was better than that of the membrane-based method. In conclusion, both preparation techniques can be used to evaluate thyroid nodules independently, particularly, the sedimentation technique can improve the preoperative detection rate of PTC.

In the present study, the differences in different LBP methods on PTC diagnosis were further evaluated from the aspects of the cell count, karyotype, and arrangement, thereby providing accurate pathological basis for independent thyroid FNAC evaluation. Ultrasound-guided FNA can allow us to obtain sufficient cells due to the accurate nodule positioning. While in this study, we found that nodules with blood flow signals bore much more cells, 87.7% (71/81) cases among which could be histologically diagnosed as thyroid malignancy, and angiogenesis was seen to exhibit intimate correlation with tumor growth, infiltration, and metastasis [12]. Among the membrane-based smears, 23.5% (36/153) cases had less than 6 clusters of follicular epithelial cells, of which 55.6% (20/36) were cytologically positive from karyotype and arrangement analysis. While among the sedimentation smears, the data were 16.2% (11/68) and 63.6% (7/11), respectively. During the pre-treatment of the specimens and the fully automated operation, cell loss might happen. Through the sedimentation technique, cells can be almost completely sedimented by gravity and collected on the slides. While in the way of the membrane-based technique, cells are diffusely tiled through high-speed rotation and air pressurization. Thus, the cell loss rate is much lower in the way of the sedimentation technique. In addition, during the sedimentation preparation process, sufficient number of cells can be obtained for cytological evaluation under the guidance of ultrasound, while during the membrane-based preparation process, more cells can be provided for aided methods after cell morphology interpretation without destroying the preserved slides, like further molecular detection.

A self-made mixture of ethanol and normal saline was once used as stationary liquid for thyroid FNAC diagnosis, finding that PTC was characterized by unobvious nuclear grooves as well as psammoma bodies and uncommon inclusion bodies [13]. Also, some studies have found that in conventional smears, true papillary tumor without PTC nucleus characteristics was considered as a benign lesion [14]. This study showed that PTC nucleus characteristics could be clearly shown in both the sedimentation and membrane-based smears, including nuclei of glass appearance, nuclear grooves, and inclusion bodies, which could help for accurate interpretation. Moreover, some structures like papillary, swirling, and syncytial structures that are common in typical papillary tumor were also could be seen. Among the membrane-based smears of PTC, 72.5% (39/131) cases had unobvious papillary or swirling structure, of which the papillary tumor was flat with unobvious three-dimensional structure. In addition, the fibrovascular axis was rare to be seen, and most cells were presented as crowded syncytial masses. While among the sedimentation smears, papillary or swirling structure could be seen in 55.2% (32/58) cases, and the papillary tumor had obvious and well-preserved three-dimensional structure; meanwhile, the fibrovascular axis still remained. Different preservation solutions for liquid-based smears have different effects on the nucleus morphology and structure of thyroid follicular epithelial cells. Knowing about these subtle differences would help us better interpret the PTC with different LBP techniques before operation. Also, different subtypes of PTC have different nucleus characteristics, so more specimens are needed for comparative studies. Especially for thyroid follicular lesions, it is very hard to be diagnosed definitely with cytology. Some studies [15] showed that among the 42 cases that were confirmed as follicle-subtype of PTC after operation, those with atypical PTC nucleus characteristics were featured with large cell count, deeply stained nucleus of the same size, uncommon nuclear grooves as well as inclusion bodies, and crowded arrangement, which might be the signs of follicle-subtype of PTC. In this study, we found that among the cases with membrane-based technique that were histologically confirmed as benign lesions, 72.7% (16/22) were cytologically interpreted as TBSIII and above classes, while in the sedimentation smears, 60% (6/10) cases were interpreted as TBSIII, showing irregular nucleus enlargement, deviated small nucleoli, nuclear grooves, crowded cell arrangement, and visible microfollicular structures. In a word, such characteristics should be carefully identified when it comes to preoperative liquid-based FNAC interpretation on thyroid nodules. Meanwhile, aided examinations like BRAFv600e and other molecular tests should be performed to avoid over-diagnosis that might cause irreversible consequences for the patients.

Due to the different compositions of the preservation solutions, the tumor cell background on the smears is different. Tumor cells in membrane-based smears are clean as the mucus and blood are pre-treated. While in sedimentation smears, it is not obvious to see the loss of non-cellular components, and inflammatory cells are more common to be seen as well as lymphocyte aggregation. Much neutrophils and histocytes could be seen in the smears by two preparation methods for nodular goiter histologically, and lymphocytes mixed with follicular epithelial cells appearing acidophilic degeneration in the smears for Hashimoto’s thyroiditis. However, these benign conditions could also be accompanied by malignant lesions, which would be mixed with peripheral normal follicular epithelial cells, bringing certain difficulties to cytological diagnosis.

In summary, this study confirmed that LBP techniques, including membrane-based and sedimentation, can be used for FNAC diagnosis in thyroid nodules. Sedimentation preparation technique was identified to be better, as it could make the PTC detection rate higher, and the cytological result based on this technique had good consistency with histopathological diagnosis after operation. Moreover, the sensitivity of the liquid-based smears in the diagnosis for preoperative PTC was higher, and the malignancy degree of nodules that were diagnosed as TBSV and TBSVI could reach 100%. In addition, PTC nucleus morphology and structural characteristics could be shown by both two LBP techniques, with papillary tumor presented to be flat in membrane-based smears and as an obvious three-dimensional structure in sedimentation smears that was easy to be identified. Therefore, better understanding the subtle differences in the evaluation of cell morphology and structure by membrane-based and sedimentation LBP techniques is conducive to the definitive diagnosis of PTC before operation.

## Data Availability

The data and materials in the current study are available from the corresponding author on reasonable request.
